# Reconstructing Demography and Social Behavior During the Neolithic Expansion from Genomic Diversity Across Island Southeast Asia

**DOI:** 10.1534/genetics.116.191379

**Published:** 2016-09-28

**Authors:** François Vallée, Aurélien Luciani, Murray P. Cox

**Affiliations:** Statistics and Bioinformatics Group, Institute of Fundamental Sciences, Massey University, Palmerston North 4442, New Zealand

**Keywords:** demographic expansion, Neolithic, Asian, Papuan, Island Southeast Asia

## Abstract

Archaeology, linguistics, and increasingly genetics are clarifying how populations moved from mainland Asia, through Island Southeast Asia, and out into the Pacific during the farming revolution. Yet key features of this process remain poorly understood, particularly how social behaviors intersected with demographic drivers to create the patterns of genomic diversity observed across Island Southeast Asia today. Such questions are ripe for computer modeling. Here, we construct an agent-based model to simulate human mobility across Island Southeast Asia from the Neolithic period to the present, with a special focus on interactions between individuals with Asian, Papuan, and mixed Asian–Papuan ancestry. Incorporating key features of the region, including its complex geography (islands and sea), demographic drivers (fecundity and migration), and social behaviors (marriage preferences), the model simultaneously tracks a full suite of genomic markers (autosomes, X chromosome, mitochondrial DNA, and Y chromosome). Using Bayesian inference, model parameters were determined that produce simulations that closely resemble the admixture profiles of 2299 individuals from 84 populations across Island Southeast Asia. The results highlight that greater propensity to migrate and elevated birth rates are related drivers behind the expansion of individuals with Asian ancestry relative to individuals with Papuan ancestry, that offspring preferentially resulted from marriages between Asian women and Papuan men, and that in contrast to current thinking, individuals with Asian ancestry were likely distributed across large parts of western Island Southeast Asia before the Neolithic expansion.

A growing body of archaeological, linguistic, and genetic evidence is increasingly clarifying the nature of population movements into and through Island Southeast Asia during the Neolithic period (Bellwood 2013). Nevertheless, much about this process remains poorly understood, particularly the dual role of social behaviors and demography in driving population movements, and how these in turn created the complex patterns of genetic admixture observed across the region today.

Computer modeling is one useful way forward. While models are always vastly—but necessarily—simpler than the real world, they allow deeper insight into the processes that produced modern patterns of genetic diversity, and notably, provide information on the interactions between those processes. Often one of their most important contributions is circumscribing what is not possible—even when models cannot distinguish between several plausible alternatives, they frequently exclude some scenarios as being inconsistent with the data. For these reasons, the use of computer simulations is now increasingly employed in anthropological settings (Kohler *et al.* 2005), with particularly sophisticated cultural models revealing social interactions within prehistoric Pueblo (Kohler *et al.* 2012) and Maya communities (Heckbert 2013). For Island Southeast Asia and the Pacific region, notable early models included Geoff Irwin’s simulations of Pacific sailing routes (Irwin 1992).

Computer simulations of human population genetic data are also illuminating key aspects of social behavior, such as admixture (Verdu, *et al.* 2013), fertility inheritance (Brandenburg *et al.* 2012), and sex-biased migration (Karmin *et al.* 2015). Many of these models use coalescent theory, a standard model framework employed in population genetics (Wakeley 2008), but alternative modeling approaches, particularly those based on explicitly simulating individuals within communities, have expanded the range of questions that can now be asked—from exploring how community connectivity is linked to the appearance of modern human behavior (Powell *et al.* 2009), the role of culturally mediated migration in driving genetic diversity within structured populations (Premo and Hublin 2009), the effects of population structure on the time to the most recent common ancestor (Rohde *et al.* 2004), and identifying how marriage rules affect patterns of genetic diversity in small traditional communities (Guillot and Cox 2014; Guillot *et al.* 2015). Individual-based models seem particularly well suited to spatially explicit simulations, with the key software in this area, SPLATCHE2 (Ray *et al.* 2010), combining forward-in-time simulation of population demography with backward-in-time coalescent modeling of genetic diversity. This approach has been employed to analyze a variety of complex demographic scenarios, including the effects of gene surfing during human range expansions (Excoffier and Ray 2008).

Many of these models are conceptual variants of agent-based modeling (Railsback and Grimm 2012), a simulation framework that is increasingly dominating complex systems research. With agent-based models, no global outcomes are programmed into the model, and broad-scale patterns instead emerge as the result of local interactions and decisions made by individual agents. Agent-based models are particularly useful because they provide near unlimited flexibility in model design, albeit at the price of strong constraints on implementation and statistical inference (Lee *et al.* 2015). Indeed, the individual-based models used in human population genetics have typically explored general theoretical expectations, rather than explicitly inferring model parameters by statistical fitting to genetic data. A notable exception is the reconstruction of global settlement history from worldwide microsatellite data (Liu *et al.* 2006).

Here, we employ agent-based modeling to reconstruct human mobility across Island Southeast Asia from the Neolithic period to the present, with a particular focus on interactions between individuals with Asian, Papuan, and mixed ancestry. Our choice of model framework reflects the complexity of regional history, including a challenging geography (a complex arrangement of islands and sea), migration at variable scales (both short and long distance mobility), and the action of social behaviors (such as sex-biased Asian–Papuan marriage preferences), all while requiring patterns of genetic diversity to be tracked simultaneously across a full gamut of marker types [autosomes, X chromosome, mitochondrial DNA (mtDNA), and Y chromosome]. Importantly, this model is not intended as an end in itself, but is instead integrated with Bayesian statistical inference to explicitly estimate demographic and social parameters that may have led to the Asian ancestry proportions observed in nuclear genetic markers across Island Southeast Asia today (Cox *et al.* 2010; Wilder, *et al.* 2011; Tumonggor, *et al.* 2014). Key questions include (i) whether incoming individuals with Asian ancestry had greater fecundity and/or propensity to migrate than local individuals with Papuan ancestry (perhaps due to improved farming and maritime technologies); (ii) whether a widely proposed bias favoring marriages between Asian women and Papuan men is required to explain increased rates of Asian variants on the X chromosome relative to the autosomes (Hage and Marck 2003; Cox *et al.* 2010); and (iii) how far individuals with Asian ancestry had encroached into western Island Southeast Asia prior to the Neolithic expansion (Spriggs 2012; Lipson *et al.* 2014).

## Materials and Methods

### Data

Reference data comprise estimates of Asian/Papuan admixture from 2299 individuals in 84 populations across Island Southeast Asia (Cox *et al.* 2010), including additional data points for North Maluku (Wilder *et al.* 2011) and West Timor (Tumonggor *et al.* 2014). Asian admixture proportions were calculated for both the autosomes and the X chromosome using 39 ancestry informative markers (AIMs), which were chosen for their high *F*_ST_ between proxy parental populations, southern Han Chinese, and Papua New Guinea highlanders. For modeling purposes, admixture values were averaged across multiple populations on small islands, and simulations were based on autosome and X chromosome admixture proportions for 16 regional groups (Supplemental Material, File S1, Table S1). Information on the design of the AIM markers, as well as access to the publicly available genetic data sets, is described in full elsewhere (Cox *et al.* 2010; Wilder, *et al.* 2011; Tumonggor *et al.* 2014).

### Agent-based model

The agent-based model was written in Java within the Repast Simphony v.2.2 framework, a widely used toolkit for supporting agent-based modeling (North *et al.* 2013). Source code for the model, together with associated documentation, including a user guide, are freely available from the model library run by the OpenABM consortium: https://www.openabm.org/model/5014.

The model simulates the mid-Holocene expansion of farming populations, ultimately from mainland Asia, across Island Southeast Asia starting 4500 years ago. Individuals with Asian ancestry encounter populations with Papuan ancestry, quickly leading to admixed individuals who carry both Asian and Papuan genetic markers, with the overall effect that Asian variants spread across the islands from west to east.

### Time

Simulations were run from 4500 years before present (BP) to the modern era, with this start time chosen to be jointly consistent with genetic (Xu *et al.* 2012), linguistic (Gray *et al.* 2009), and archaeological evidence (Spriggs 2011). The model progresses in time steps of 1 year, providing a balance between simulation speed and approximation to reality (for instance, individuals in the real world typically do not give birth or marry more than once per year).

### Agents

**Figure 1:**
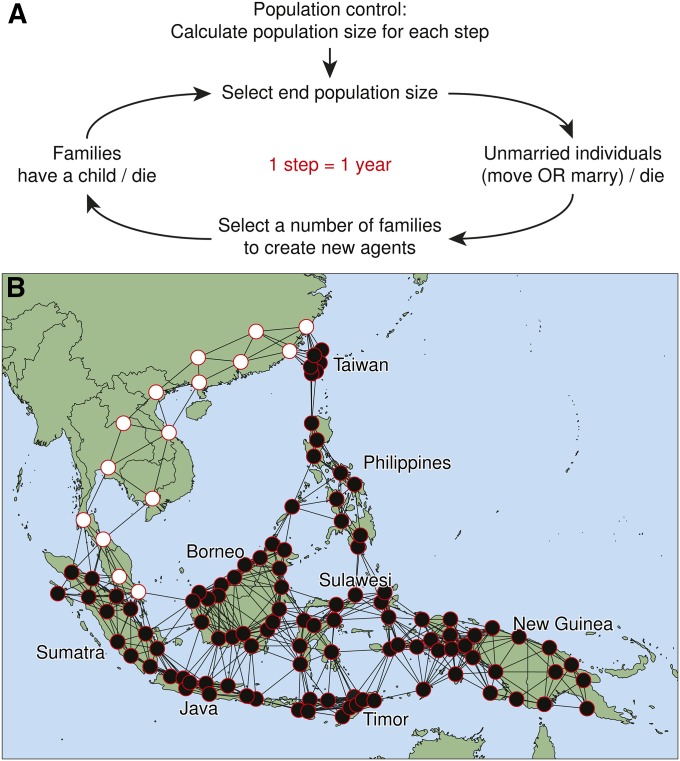
Overview of the structure of the agent-based model system. (A) Summary of the life cycle of individuals, showing the actions of individuals (such as birth, migration, marriage, and death) and actions of the model environment (such as control of population growth and the creation of families). (B) The population network structure, showing just one of 82 starting distributions for Asian (white) and Papuan (black) populations.

The model comprises individuals (“agents”) who are born, may move to a neighboring community, can marry, have children, and die (Figure 1A). The concept of “computer people” is therefore a close fit to the underlying algorithm, rather than just being an apt analogy. Agents fall into two classes: unmarried individuals and families. Unmarried agents mimic real individuals: they are either men or women, they carry genetic markers (which determine whether an agent is defined as Asian or Papuan), and they are mostly young (between 0 and 15 years old), although older individuals can occur. When an agent reaches maturity (defined as 18 years old), they can perform two new actions: move or marry. A random variable is drawn to determine whether an unmarried individual moves, marries, or remains in an unmarried state, with only one action allowed within a single time step (Figure S1A).

An agent can either stay within its natal village or move to an adjacent community, but agents cannot move more than once in their lifetime and not at all after they marry. This mimics a salient aspect of Island Southeast Asian prehistory, where even today, individuals still mostly live in small traditional groups and typically only move to marry, usually to a neighboring community (Guillot *et al.* 2015). Agents can move to any community within a radius of 650 km, but movements to nearby communities are strongly favored. Potential recipient communities are ranked by distance, a random value is drawn from a β-distribution (Table S2), and the proportionally closest index to this value is selected as the recipient community. This process thus captures both (i) frequent marriage into neighboring groups and (ii) rarer long-distance “leapfrog” movements, which are increasingly viewed as a defining feature of Island Southeast Asian settlement (Spriggs 2012). Agents can only move to a community that is not at carrying capacity.

To marry, an agent must have reached maturity and must find a partner no more than 6 years younger or older, an age range suggested from cross-cultural studies (Fenner 2005). While most agents marry, marriage may not occur if an appropriate partner cannot be found. Marriages fall into four classes, which by default occur with equal probability (Table 1): within group marriages (Asian men marrying Asian women, and Papuan men marrying Papuan women), and between group marriages (Asian men marrying Papuan women, and vice versa). A marriage weighting parameter (*M*) favors marriages between Papuan men and Asian women, which has been proposed to explain greater Asian ancestry on the X chromosome compared to the autosomes (Cox *et al.* 2010; Lansing *et al.* 2011). *M* takes values in the interval [0, 0.25], ranging from no preference (0) to strong preference (0.25) for marriage between Asian women and Papuan men. Because there are four possible classes of marriages, values >0.25 are functionally equivalent to this upper bound.

**Table 1:** Marriage rates as modified by the weighting parameter *M*

	Asian men	Papuan men
**Asian women**	0.25	0.25 + *M*
**Papuan women**	0.25 − *M*	0.25

On marriage, two individual agents merge to form a family agent. Each family is randomly assigned a maximum allowable number of children, drawing on a random value from a Poisson distribution for fecundity, which differs for Asian and Papuan mothers. A lower bound of 3.5 was set for two reasons: first, to prevent bouts of community extinction that were commonly observed at lower values, even though these are not seen in the real world; and second, because a recent cross-cultural study (Jones and Tuljapurkar 2015) considered this to be a lower limit on fecundity for Neolithic groups. Births were only allowed when the population was not at carrying capacity.

Agents carry a suite of AIMs, which they pass on to their offspring, thus closely mimicking the real genomic data set (Cox *et al.* 2010). When a new agent is created (*i.e.*, a child is born), their genome is constructed from the two parent genomes (Figure S1B), with genetic markers transmitted according to the usual biological rules. Markers on the autosomes (*n* = 25) and the X chromosome (*n* = 25) are implemented as binary arrays, with 0 and 1 indicating Papuan and Asian alleles. Autosomal markers are treated as completely unlinked (equivalent to the real AIM data set) and are therefore picked randomly from each parent. X chromosome markers are partially linked, and contiguous blocks of markers are chosen based on recombination breakpoints simulated using the X chromosome recombination rate (International HapMap Consortium 2003; McVean *et al.* 2005). As an approximation to the original genetic data set (Cox *et al.* 2010), X chromosome markers in the model are distributed uniformly along the X chromosome. All markers on the Y chromosome and mtDNA are fully linked, and so for the purposes of determining ancestry, each is represented internally as just a single binary variable. A proxy for ethnicity is derived from these markers: an individual is defined as Asian if ≥50% of its genomic markers have Asian ancestry. An alternative definition, under which individuals are treated as Asian if they have at least one Asian ancestor, produced simulations with poor fits to the observed genetic data. Because every individual has many ancestors, the simulations rapidly converged to the point where most agents had at least one Asian ancestor, even if only distantly.

### Demography

Mortality rates were taken from the closest regional and temporal data set, an analysis of Taiwanese populations in 1970 (University of California Berkeley and Max Planck Institute for Demographic Research 2000). Because these reflect modern rather than traditional societies, death rates were rescaled from ages 0–108 to 0–55 to mimic the age distribution of regional communities during the Neolithic (Wang 2008) and doubled to capture the higher death rates of traditional farming groups suggested by cross-cultural studies (Fenner 2005) (Figure S2). Birth rates vary by age and were taken from a population study in the United States in 1940, prior to effective birth control measures (National Center for Health Statistics 2016) (Table S3). Mortality and birth rates are intended only as reasonable proxies for unknown values, but both distributions have characteristic curves with relatively limited variability across many human populations and simulations were insensitive to exact values.

Age classes were used to initialize populations at the start of each simulation, using empirical archaeological data from regional Neolithic cemetery sites. Numerical values for classes were set from a Neolithic population along the Yellow River in China (9000–3500 BP) (Wang 2008), but these closely resemble those at the early Lapita site of Teouma in Vanuatu (3200–3000 BP) (Bentley *et al.* 2007) at the other end of the geographical range of the model. Due to limitations in estimating age from osteological measurements, these studies provide age distributions only within broad brackets. The model was initialized with the age structure estimated for the Yellow River community: 30% children (0–15 years old), 30% youth (16–25 years old), 30% prime age (26–35 years old), and 10% middle age (36–50 years old). Within the first generation, this age profile shifts slightly younger to 40% children, 25% youth, 25% prime age, and 10% middle age, thereafter remaining stable throughout the simulation.

To match the weak population growth inferred for communities across Island Southeast Asia (Guillot *et al.* 2013), demes were initialized with 120 individuals, at the low end of estimated community sizes today (Lansing *et al.* 2008). The carrying capacity was capped, but increased exponentially over time, with population dynamics free to fluctuate below this limit (Table S2).

### Environment

Island South East Asia is a special landscape, with a mosaic of sea and islands that are diverse in shape, size, and topology. In the model, agents populate a network of demes (Figure 1B), whose number, distribution, and connections are defined by the size of each island. For computational constraints, we modeled 116 demes (101 within the islands, or one every 6000 km^2^) (Table S4). As larger islands typically contain underpopulated mountainous interiors, fewer demes were assigned to large islands, such as Borneo, and demes were preferentially placed along the coasts. Nevertheless, larger islands still have larger networks and more connections than smaller islands. To facilitate statistical inference, demes were preferentially placed on islands where real genomic information was available. Mainland Asian populations are treated as special “source” demes and therefore purposely do not scale with land area. Connections between all demes were determined by applying a 650-km threshold using great circle distances, thus permitting both nearby demic movements and larger leapfrog dispersals. Movements over land and sea were not differentiated, although mobility may have been mediated along voyaging corridors. Greater migration by sea can lead to faster population spread (Figure S3), although interactions with birth rates, population growth, and carrying capacity suggest that this outcome is far from straightforward. Addressing this issue further was considered beyond the statistical power of the current genetic data set.

At the beginning of each simulation, demes contain individuals carrying only Asian or Papuan markers. The initial distribution of individuals with Asian ancestry (*D*) was inferred from a large set of starting distributions (*n* = 82). Some distributions restricted Asian individuals to the mainland (Figure 1B), consistent with evidence from physical anthropology that supports the concept of an “Old Melanesia,” which once spanned from mainland Asia to New Guinea (Howells 1976), while other starting distributions had Asian populations inhabiting parts of western Island Southeast Asia, as perhaps indicated by recent genetic surveys of haploid loci (Karafet *et al.* 2010; Tumonggor *et al.* 2013) and the autosomes (Lipson *et al.* 2014). The starting distributions fell into four broad classes, with Asian populations initially dispersed across (i) just mainland Asia, (ii) mainland Asia plus northern Island Southeast Asia (Taiwan and/or the Philippines), (iii) mainland Asia plus western Island Southeast Asia (Sumatra, Java, and/or Borneo), or (iv) mainland Asia plus northern and western Island Southeast Asia. Intermediate variants were obtained from the last three classes by adding Asian nodes in groups of four demes to sequentially cover Java, Borneo, and the Philippines in multiple permutations, thus creating the 82 distributions tested. Asian populations were constrained to be adjacent (thus excluding nonclustered random distributions) and historically unsupported scenarios were not explored (for instance, models with Asian populations in the islands and Papuan populations on the mainland).

### Model parameters

Due to the complexity of the model system and its relatively low run-time speed (each simulation takes ∼2 min parallelized on a 6-core computer with a 3.07 GHz Intel Xeon processor), statistical power limits inference to only a small number of parameters (Table S2). Two have already been described: the marriage weighting parameter (*M*) and initial distribution of individuals with Asian ancestry (*D*). In addition, the rapid spread of individuals with Asian ancestry might result from either (i) increased fecundity or (ii) increased mobility relative to individuals with Papuan ancestry. Therefore, four additional parameters were inferred: the probability that an Asian individual migrates to a new community to marry (*m*_A_), the probability that a Papuan individual migrates to a new community to marry (*m*_P_), the fecundity of Asian individuals (*f*_A_), and the fecundity of Papuan individuals (*f*_P_). Migration probabilities have a theoretical range of 0–1, but were constrained here to [0.1, 0.8] because preliminary testing showed that simulations under lower and higher values produced poor fits to the observed genetic data. Fecundity values (Poisson means) range from 3.5 to 7, as suggested from a cross-cultural study of fecundity in small traditional human groups (Jones and Tuljapurkar 2015) and preliminary model testing.

### Statistical inference

Although populations are initially either Asian or Papuan, individuals quickly arise who carry genomic markers with both ancestries, but at different proportions across the geographical space. The mean proportions of Asian ancestry on the autosomes and X chromosome for simulated populations can be related directly to the admixture proportions observed for real human groups: here, AIM data for human populations distributed across Island Southeast Asia (Cox *et al.* 2010; Wilder *et al.* 2011; Tumonggor *et al.* 2014). Model parameters were then inferred by minimizing the fit between the simulated and real ancestry values.

The model returns the Asian ancestry proportion, pooled across markers on both diploid chromosomes for all simulated individuals, separately for the autosomes and X chromosome:

$$\text{Asian Ancestry }\left(\text{Individual}\right)=\frac{\text{Number of Asian Markers}}{\text{Total Number of Markers}}$$

which can then be used to calculate average Asian ancestry proportions for populations:

$$\text{Asian Ancestry }\left(\text{Population}\right)=\frac{\Sigma \text{Asian Ancestry }\left(\text{Individual}\right)}{\text{Population Size}}$$

Although the variance of ancestry proportions among individuals within a population can be useful for reconstructing admixture processes (Verdu and Rosenberg 2011), mean ancestry per population was used for fitting here because ancestry calculated from AIM data sets appears to dampen variation among individuals (Wilder *et al.* 2011), while remaining robust at the population level (Cox *et al.* 2010). The model was fitted only for the autosomes and X chromosome because ancestry assignments are uncertain for some mtDNA and Y chromosome haplogroups (Karafet *et al.* 2010; Tumonggor *et al.* 2013). Comparisons of ancestry on the haploid loci were instead used as a downstream validation check. Estimates of Asian ancestry on the autosomes and X chromosome were modeled for 16 regional groups by combining multiple populations for small islands, and these 32 values were used as summary statistics for parameter inference within an approximate Bayesian computation (ABC) setting.

The final simulation data set comprised 500,000 runs taking ∼1120 days of compute time. Inference of continuous parameters (*i.e.*, excluding the discrete initial Asian distributions), together with statistical cross-validation checks and the calculation of prediction errors (*E*_pred_), was undertaken using the R package abc v.2.1 (Csilléry *et al.* 2012). The optimal tolerance value (0.01) was estimated by minimizing *E*_pred_ values using a standard leave-one-out cross-validation procedure, also as implemented in abc. Optimal parameter estimates were obtained using local linear regression, but a full range of alternative statistical methods, such as rejection and neural networks, was also run. The initial distribution of Asian populations was estimated by calculating the frequency of each of the starting distributions in the final set of accepted simulations.

### Data availability

The authors state that all data necessary for confirming the conclusions presented in the article are represented fully within the article.

## Results

### Data validation

To confirm that ancestry estimates from AIM data are robust, maximum likelihood estimates of Asian ancestry were inferred using ADMIXTURE v.1.30 (Alexander *et al.* 2009) from unpublished autosomal Affymetrix SNP array data (548,994 markers) for 323 individuals from 15 paired, but not directly overlapping, populations (Figure S4). Because Asian–Papuan ancestry is the primary signal in the data (Sanderson *et al.* 2015), ancestry estimates were inferred at *K* = 2. Population means of Asian ancestry inferred from the AIM and SNP array data are highly correlated (*r* = 0.99, *P* ≪ 0.001) (Figure S4), suggesting that the geographically more extensive AIM data set (84 *vs.* 15 populations) is robust for modeling purposes.

### Model validation

Because a new model was developed, it was critical to assess its reliability and the reproducibility of its results. This was undertaken through two standard validation approaches in agent-based modeling: stability and sensitivity testing. Stability analyses check that simulations produce similar outputs when run with the same set of input parameters. However, as simulations run with many random number draws, they should still exhibit stochastic variation, a characteristic feature of all real-world biological systems. Simulations for this model produce results that are tightly bounded around their means for a given set of parameters, while still showing small levels of variance (Figure S5 and Figure S6). In contrast, sensitivity analyses check whether invariant parameters have substantial effects on model behavior. For instance, preliminary testing emphasized that population size and growth rates must be bounded, otherwise populations rapidly explode in size or collapse (Geard *et al.* 2013), even though these behaviors are not routinely observed at this scale among human populations in the real world. Beyond extreme values, sensitivity testing shows that fixed parameters (such as population size and growth, birth, and death rates) have little influence on model results.

**Figure 2:**
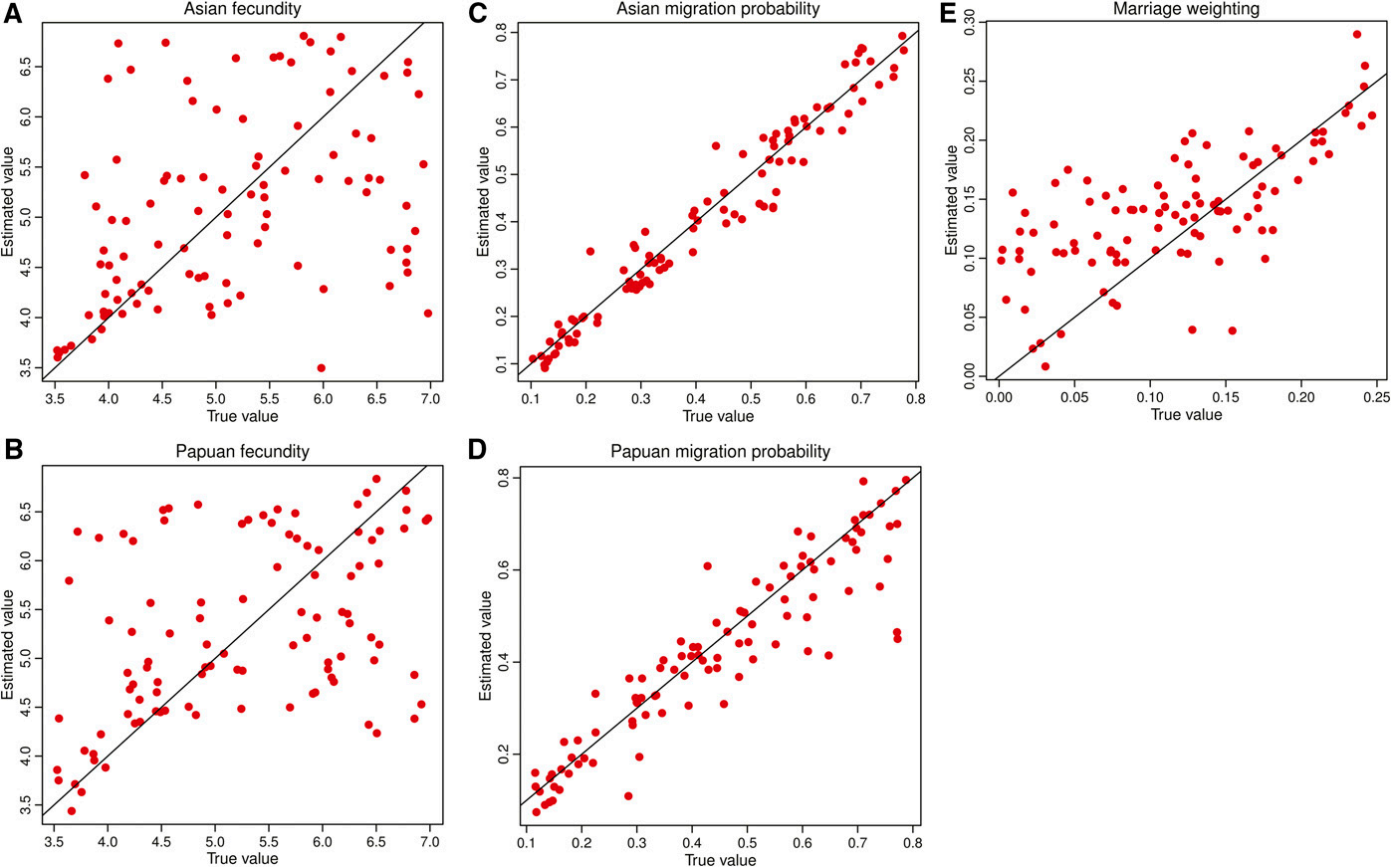
Testing the accuracy of the inference procedure. Cross-validation results are shown for 500,000 simulations, illustrating variable ability to infer five model parameters: (A) Asian fecundity, (B) Papuan fecundity, (C) Asian migration probability, (D) Papuan migration probability, and (E) marriage weighting.

Parameter inference was performed within an ABC setting (Beaumont *et al.* 2002) and validated using a standard cross-validation approach (Csilléry *et al.* 2010). Estimated prediction errors show the extent to which known, but blinded, parameters from randomly chosen simulations can be inferred. Prediction errors were minimized at a tolerance value of 0.01, and vary from *E*_pred_ = 0.05 to 1.08. Greatest statistical power was found to infer migration probabilities: *E*_pred_(*m*_A_) = 0.16 and *E*_pred_(*m*_P_) = 0.05, followed by the marriage weighting: *E*_pred_(*M*) = 0.66, with less power to infer rates of fecundity: *E*_pred_(*f*_A_) = 0.98 and *E*_pred_(*f*_P_) = 1.08. (Prediction errors require continuous or ordinal variables, thus precluding their application to the discrete, but categorical, starting distributions). The fit between known and estimated values across 500,000 simulations (shown graphically in Figure 2) shows that the model has variable, but generally good, statistical power to infer the model parameters.

Two additional checks were performed to validate basic demographic behavior. First, demes were evaluated for aberrant demography by tracking the maternal ages at which births occurred and the ages of all agents at their deaths. As expected, the distribution of ages when mothers gave birth and agents died fit the modeled birth (Table S3) and death rates (Figure S2). Second, the age of mothers at their first and last births was compared against estimates from the Neolithic site in the Yellow River Valley (Wang 2008). While the archaeological data have limited resolution, the distributions were qualitatively similar.

### Relative importance of summary statistics

Some summary statistics are more informative than others; for instance, a small number of nodes near the Asian mainland always reached 100% Asian ancestry, and by exhibiting zero variance, these provide no information to discriminate between different values of the model parameters. Adjacent populations also routinely show partially correlated levels of Asian ancestry, and some of these summaries could likely be dropped without substantially changing the inference. We note, for instance, that Asian admixture proportions on Sumatra and Java, two neighboring islands, are frequently similar. For simplicity, however, all summary statistics were retained in the analysis.

### Parameter inference on real data

**Figure 3:**
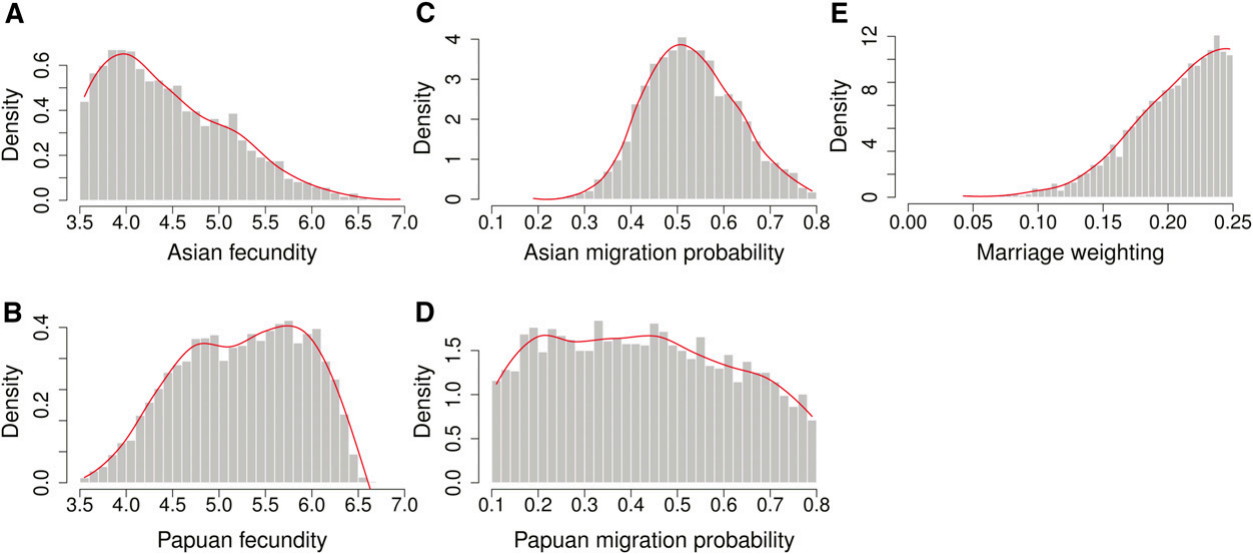
Histograms of Bayesian posterior densities for model parameters. (A) Asian fecundity, (B) Papuan fecundity, (C) Asian migration probability, (D) Papuan migration probability, and (E) marriage weighting. Red lines show local linear regression smoothing.

The model was used to infer migration, fecundity, the marriage weighting, and the initial distribution of Asian ancestry by fitting to Asian ancestry proportions calculated from a genomic data set of populations across Island Southeast Asia (Cox *et al.* 2010; Wilder *et al.* 2011; Tumonggor *et al.* 2014). By retaining the set of simulations with the smallest Euclidean distances relative to Asian ancestry proportions in the observed data, estimates can be made of the model parameters (Figure 3). The probability of moving into a new community to marry was found to be nearly twice as large for Asian individuals [0.51, 95% credible region (CR): 0.36–0.75] as Papuan individuals (0.31, 95% CR: 0.10–0.80), while fecundity trended lower for Asian individuals (3.9, 95% CR: 3.4–5.9) compared to Papuans (5.7, 95% CR: 3.9–6.3). However, neither difference is statistically significant. The marriage weighting was estimated at 0.23 (95% CR: 0.11–0.25), excluding the “no preference” case at M = 0, thus showing clear support for preferring offspring from marriages between Asian women and Papuan men. (Note that simulations with a marriage weighting >0.25 are indistinguishable from simulations at 0.25).

As migration and fecundity both act as pressures for spreading Asian alleles, it was hypothesized that these variables might be negatively correlated. An association between migration and fecundity explains 29% of the variance for Asians (*r* = −0.54, *P* ≪ 0.001), but no meaningful association occurs for Papuans (*r* = 0.027, NS) (Table S5). Higher migration and higher fecundity in Asians therefore both act as drivers for creating the observed patterns of Asian–Papuan ancestry across Island Southeast Asia, although other factors must also be in play. Other pairs of parameters exhibit weaker associations, with only half as much variance explained by the next largest association (Figure S7). A key message is that migration and fecundity are interconnected drivers that appear to interact in surprisingly complex ways.

**Figure 4:**
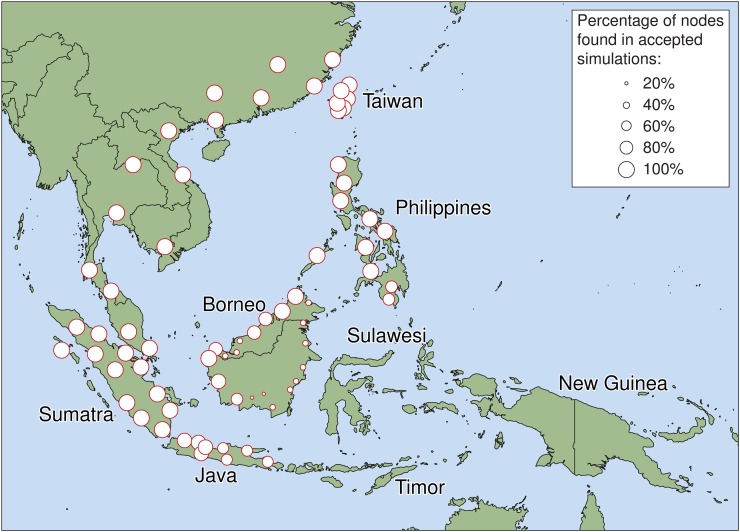
Bayesian posterior density of nodes showing the likely initial distribution of individuals with Asian ancestry prior to the mid-Holocene Neolithic expansion. Node sizes are plotted relative to their frequency in the set of accepted simulations. The inset shows representative circle sizes for reference.

To determine the pre-Neolithic distribution of populations with Asian ancestry, simulations were run with 82 starting distributions of Asian nodes, ranging from restricting Asian groups to the mainland, to Asian populations being dispersed across parts of Taiwan, Sumatra, Java, Borneo, and the Philippines. Only 32 of these distributions were represented in the final subset of simulations with a close fit to the genomic data (Figure 4). These illustrate strong support for Asian individuals being distributed widely across western parts of Island Southeast Asia prior to the main Neolithic expansion.

### Further model confirmation

To validate the behavior of the model under the parameter values with highest probability (Figure 3), we sought to confirm that three key features of the real world data also appear in the simulated results. First, diploid loci (autosomes) exhibit lower variance in Asian admixture proportions than haploid loci (mtDNA and the Y chromosome), due to their fourfold higher effective population size, as well as an admixture rate averaged over a larger number of unlinked markers (Figure S5). Second, simulated data exhibit the characteristic “step-like” pattern of greatest Asian ancestry on the mtDNA, followed by the X chromosome, then the autosomes, and finally the Y chromosome, resulting from either a bias toward marriages between Asian women and Papuan men, or preferential survival of offspring from such marriages (Lansing *et al.* 2011) (Figure S6). Third, the Neolithic dispersal occurs at similar rates in the model and the real world. Rates of spread were estimated by recording the time step when (i) the first Asian marker or (ii) the first Asian agent appeared in each deme, with rates normalized by the distance between demes. The estimated rate of spread of Asian markers in 200 simulations under the most probable set of parameter values was 11.5 km/year [95% confidence interval (C.I.) (7.83, 28.7)], with the estimated rate of spread of Asian agents ∼4.00 km/year [95% C.I. (1.44, 18.6)]. Literature values vary from 0.9 (±1.3) km/year estimated from genetic data (Xu *et al.* 2012), to 3 km/year estimated from archaeology (75 km/25 year generation; Bellwood *et al.* 1995), to 6.5 km/year estimated from linguistic data (Gray *et al.* 2009), all providing a reasonable fit with the rate at which Asian agents spread in the model. Note that this is the rate at which individuals move to a neighboring community to marry, and has little connection with how far individuals can move on a day-to-day basis (which might be 10–20 km/day on foot and 50–150 km/day on an outrigger canoe; Bellwood *et al.* 1995). The critical parameter is the rate at which people move to resettle and marry. In summary, the optimal parameters inferred under this model produce simulations that closely match key features of the Neolithic dispersal that occurred across Island Southeast Asia.

## Discussion

Agent-based modeling, a simulation framework with limited prior use in population genetics, was employed here to simultaneously infer demographic and social features of the Neolithic expansion across Island Southeast Asia. The model purposely does not aim for perfect realism, but instead captures salient aspects of the Neolithic dispersal. In particular, it mimics the complex geography of Island Southeast Asia, with its patchy distribution of islands and sea, as well as a preference for coastal habitation, as opposed to more sparsely populated highlands. It simulates a range of starting distributions for Asian genomic ancestry at the beginning of the Neolithic period, with Asian lineages either restricted to the mainland or dispersed across western Island Southeast Asia. It also implements a hypothesized social preference for marriage between Asian women and Papuan men and simulates key drivers of demic spread, including variable birth rates and propensity to migrate. Crucially, these behaviors can vary between groups with different ancestry states, and depending on marriage choices, genomic ancestry can change radically between generations, even along a single family line. For study systems with similarly striking levels of complexity, agent-based models promise to be a useful, if currently underutilized, model framework.

Model parameters were fitted to genomic data from 2299 individuals in 84 populations across Island Southeast Asia, spanning Taiwan in the north, Sumatra and Java in the west, and New Guinea in the east. Simulations under the optimal parameter set (see video in File S2) produce genomic data that closely match real distributions of Asian ancestry across the region, providing some indication of the conditions that may have prevailed in the past. The model therefore acts as at least a reasonable proxy for major aspects of the population dispersal that occurred across Island Southeast Asia during and following the Neolithic period. On these grounds, the model was used to address three major questions.

### Did individuals of Asian and Papuan descent differ in birth rates and propensity to migrate?

A general premise is that incoming groups with ultimate Asian ancestry had two primary advantages that drove their expansion into Island Southeast Asia: first, they had better sailing technologies, allowing them higher rates of migration (Bellwood *et al.* 1995); and second, they had improved farming practices, offering a higher effective birth rate (likely through greater survivorship of children) (Shennan *et al.* 2013). The modeling results are largely agnostic on these points. The probability with which Asian individuals migrate falls within relatively tight bounds (mode 0.51, 95% CR: 0.36–0.75) and is higher but statistically indistinguishable relative to Papuan individuals (0.31, 95% CR: 0.10–0.80). Nor is there a statistically significant difference in birth rates between Asians (3.9, 95% CR: 3.4–5.9) and Papuans (5.7, 95% CR: 3.9–6.3). If anything, Asian birth rates trend slightly lower, although of all the parameters modeled, the least statistical power is available to infer fecundity. Demes on mainland Asia are explicitly modeled as source populations, thus providing an intrinsic pressure for outbound Asian movements. Nevertheless, Asian individuals do not show a clear advantage in either birth rates or propensity to migrate, suggesting that these behaviors may not be a strict requirement for producing admixture patterns like those observed across Island Southeast Asia today (or alternately, that very small, but cumulative, differences may be what is important).

### Were marriages favored between Asian women and Papuan men?

A growing body of evidence (Hage and Marck 2003; Cox *et al.* 2010) notes that Asian variants appear more frequently in female sex-linked regions of the genome. Markers with Asian ancestry are therefore most common on the mtDNA (inherited only through women), then on the X chromosome (which spends two-thirds of its time in women and only one-third in men), then the autosomes (equal time in men and women), and finally on the Y chromosome (inherited only through men) (Lansing *et al.* 2011). This unexpected pattern suggests that marriage must have been strongly favored between Asian women and Papuan men and/or that the offspring of such marriages had a social or biological advantage. No consensus on the causes of such a bias has yet emerged. It may relate to the prevalence of matrilocality during the early spread of Asian Austronesian-speaking communities (Jordan *et al.* 2009)—on marriage, women stayed put, instead importing husbands from neighboring communities. Other social drivers may have acted too, such as women improving their access to local resources, or biological causes, such as sex-linked variants providing resistance to local diseases, although relatively few genes occur on the X and Y chromosomes (Lander *et al.* 2001). While these possibilities remain speculative, the model nevertheless confirms the bias: a marriage weighting of zero (that is, no preference for marriages between Asian women and Papuan men) can be statistically excluded. Instead a strong weighting is required to create admixture patterns resembling those in the observed genomic data. While the model provides no additional insight into the social or biological causes of this marriage preference, it does emphasize that this bias is an important, but poorly understood, feature of the Island Southeast Asian Neolithic migration process. Understanding this feature better will likely be necessary for a full appreciation of the dynamics of population movements and interactions across this region.

### Were Asian populations present in Island Southeast Asia before the Neolithic expansion?

The distribution of individuals with Asian ancestry prior to the Neolithic is an open question. The earliest human remains from western Island Southeast Asia, such as the “deep skull” at Niah Cave in Borneo (Krigbaum and Datan 2005), seem phenotypically Australo-Papuan, suggesting that groups with Papuan affinity were once more widely dispersed than at present. Howells characterized this region as Old Melanesia, suggesting that individuals with Papuan ancestry were once distributed widely across Island Southeast Asia, but have since been pushed to the east by expanding Asian groups, leaving behind only relict Negrito populations (Howells 1976). It is unclear when this switch might have taken place: either during the Neolithic or earlier during the late Pleistocene.

The modeling strongly supports simulations with Asian populations initially distributed across large parts of western Indonesia, including Sumatra, Java, and Taiwan, as well as parts of Borneo and the Philippines (Figure 4), which may be consistent with new genetic results. Genome-scale SNP array data (Lipson *et al.* 2014) suggest that at least two Asian ancestry components are present in Island Southeast Asia: the first distributed widely, with northerly connections (southern China and Taiwan), and presumed to reflect the Neolithic north-to-south expansion of groups speaking Austronesian languages (Bellwood 2005); and a second previously unrecognized genetic component mostly restricted to western Island Southeast Asia, but associated with populations on the adjacent mainland, such as Vietnam. The current distribution of these “mainland Asian” lineages in Sumatra, Java, and Borneo (Lipson *et al.* 2014) is a surprisingly close fit to the modeling results obtained here.

There are two potential explanations for this Asian genomic component in western Island Southeast Asia. First, it may reflect a second Neolithic expansion event. Paddle-impressed pottery in Sumatra and western Java have putative mainland connections (Spriggs 2012) and there are indications of linguistic substrata in western Indonesian languages too—Austronesian languages share words (including “dog”) with Austro-Asiatic and basic numerals with Tai-Kadai (Blench 2012). Alternately, this connection may reflect late Pleistocene movements, particularly as Sumatra, Java, and Borneo were joined to the mainland during glacial periods when sea levels were much lower. The late Pleistocene Hoabinhian industry is found from the north coast of Vietnam to northern Sumatra, and variants may reach as far east as Borneo, Sulawesi, and the Philippines (Moser 2012). Presumably other human groups also entered western Island Southeast Asia from the mainland during the 45,000 years from the region’s first settlement until the Neolithic period, and it seems conceivable that they may have brought at least some of this mainland Asian genomic component with them. The modeling performed here hints at this option. Two broadly contemporary Neolithic expansions would closely resemble the “mainland only” starting distribution of Asian populations as implemented in the simulation model, but this produces genetic patterns with a poor match to real-world genetic observations. Instead, the model favors scenarios where individuals with Asian ancestry already dominated large parts of western Island Southeast Asia, at least by 4500 BP. Note that this does not exclude additional movements from mainland Asia during the Neolithic period—secondary spreads would fit well with aspects of the archaeological and linguistic evidence discussed above.

### Conclusions

We describe a new computer model that can be used to explore human movements and interactions in Island Southeast Asia from the Neolithic to the present. While purposely not attempting to simulate the full complexity of Island Southeast Asian prehistory (due to strong computational and statistical constraints), the model instead captures key features of the region, simulating genetic data under a subset of model parameters that are a close fit to observed patterns of real genomic diversity. Future benefits might accrue from implementing more complex model features, such as alternative household structures, as opposed to individual *vs.* family agents (Geard *et al.* 2013); more subtle population subdivisions, such as those now arising from genome-scale population studies (Lipson *et al.* 2014); a wider variety of migration mechanisms, such as explicitly distinguishing short- *vs.* long-range mobility (Spriggs 2000); and better discrimination of movements over land *vs.* sea. Some regional features of population demography, such as inbreeding (endogamy), might reduce marriages with neighboring communities and lead to slower rates of spread, while other features, such as strong bottlenecks, might produce more rapid changes in allele frequencies and hence sharper admixture boundaries. Our current data set lacks sufficient information to infer this wider range of parameters, and how these and other features of population demography might interact to affect genomic ancestry across geographical space is therefore not known. More complex models may be possible as extensive genome-wide data become available, thus permitting the use of more powerful descriptors of the admixture process, such as the variance in admixture proportions among individuals (Verdu and Rosenberg 2011) and the distribution of admixture block sizes (Sanderson *et al.* 2015). Nevertheless, current results already provide new insight into the prehistory of the region, suggesting that migration and fecundity had interconnected roles in driving the expansion of individuals with Asian ancestry, that Asian women had a strong preference for marriages with Papuan men, and that individuals with Asian ancestry were likely distributed across parts of western Island Southeast Asia before the Neolithic expansion.

### Software

The source code for the agent-based model, with associated documentation, is freely available from the model library run by the OpenABM consortium: https://www.openabm.org/model/5014.
